# Pulmonary ventilation images from planning CT images using deep learning on locally‐advanced NSCLC patients

**DOI:** 10.1002/acm2.70777

**Published:** 2026-08-28

**Authors:** Yoshiyuki Katsuta, Taichi Hoshino, Takaya Yamamoto, Noriyuki Kadoya, Shohei Tanaka, Kazuhiro Arai, Rei Umezawa, Noriyoshi Takahashi, Keiichi Jingu

**Affiliations:** ^1^ Department of Radiation Oncology Tohoku University Graduate School of Medicine Sendai Japan

**Keywords:** lung cancer, radiotherapy, ventilation images

## Abstract

**Background:**

Pulmonary ventilation imaging has become an increasingly important component in thoracic radiotherapy, as it is applied to functional avoidance radiotherapy, where radiation doses are strategically minimized to preserve functional lung regions.

**Purpose:**

We developed a framework that generates computed tomography ventilation images (CTVI) from planning CT (PCT) images and demonstrated its estimation performance.

**Methods:**

The subjects were a patient cohort consisting of 102 locally‐advanced non‐small‐cell lung cancer (NSCLC) patients who received radiotherapy from 2014 to 2023. The PCT images were acquired while ensuring the absence of baseline drift, frequency variation, amplitude changes, and additive random observation noise using a real‐time position management (RPM) system to minimize the introduction of inaccuracies into PCT images. On the day of PCT scan, CTVI based on four‐dimensional computed tomography images (${\mathrm{CTVI}}_{4\mathrm{DCT}}^{\mathrm{DIR}}$) images was also generated using both deformable image registration and the computing methods employed in the VAMPIRE study.

**Results:**

The CTVI based on PCT (${\mathrm{CTVI}}_{\mathrm{PCT}}^{\mathrm{DNN}}$) estimated by a model trained via hyperparameter optimization of a U‐net deep neural network with 5‐fold cross‐validation was compared with ${\mathrm{CTVI}}_{4\mathrm{DCT}}^{\mathrm{DIR}}$. In 5‐fold cross‐validation, the average voxel‐wise Spearman's correlation coefficient (*r*_s_) ± one standard deviation between ${\mathrm{CTVI}}_{\mathrm{PCT}}^{\mathrm{DNN}}$ and ${\mathrm{CTVI}}_{4\mathrm{DCT}}^{\mathrm{DIR}}$ was 0.77 ± 0.08. The Dice similarity coefficient (DSC) was computed for three functional regions (high, moderate, and low), each delineated by approximately equal volumes, obtaining DSC_high_, DSC_moderate_, and DSC_low_ values of 0.68 ± 0.06, 0.51 ± 0.08, and 0.75 ± 0.04, respectively.

**Conclusions:**

We successfully developed a framework that estimates ${\mathrm{CTVI}}_{\mathrm{PCT}}^{\mathrm{DNN}}$, and demonstrated its estimation performance in terms of Spearman's correlation coefficient and DSC on locally‐advanced NSCLC patients.

## 1 INTRODUCTION

Pulmonary ventilation imaging has become an increasingly important component in thoracic radiotherapy. A critical element of pulmonary ventilation imaging is functional avoidance radiotherapy, where radiation doses are strategically minimized to preserve functional lung regions.^1^ Traditionally, functional lung regions can be displayed on ventilation images relied on modalities, for example, positron emission tomography (PET)^2^ and single‐photon emission computed tomography (SPECT).^3^ These modalities provide valuable information in thoracic radiotherapy; however, their clinical utility is frequently constrained by practical limitations, including low resolution, additional costs, for example, radioactive tracers, exposure, prolonged scan, and long processing times. In contrast, novel ventilation image that proposed by integrating four‐dimensional CT (4DCT) images into CT ventilation images (CTVI) can help address the practical limitations of the PET and SPECT modalities.^4^

A CTVI estimated from a 4DCT image (${\mathrm{CTVI}}_{4\mathrm{DCT}}^{\mathrm{DIR}}$), which is created by combining 4DCT images and deformable image registration (DIR) technology, can be separated into two types according to the image processing method. The first method, which is referred to as the Hounsfield unit (HU) metric, involves changes in the HU in the corresponding voxel between the registered inhalation and exhalation phases in 4DCT images.^5^ The second method involves a Jacobian metric that utilizes spatial deformation information, which reflects local volume changes associated with respiratory motion.^6^ These computational strategies have been validated and demonstrate promising results in generating ventilation images that are comparable to those obtained using conventional imaging modalities,^7^ thereby reinforcing the clinical utility of ${\mathrm{CTVI}}_{4\mathrm{DCT}}^{\mathrm{DIR}}$ in thoracic radiotherapy.^8^, ^9^, ^10^, ^11^

Recently, several studies have identified a practical limitation regarding the high dependency of displaying functional lung regions in ${\mathrm{CTVI}}_{4\mathrm{DCT}}^{\mathrm{DIR}}$ on the accuracy of DIR.^12^, ^13^ Focusing on this need to reduce reliance on DIR, recent advances in convolutional neural networks (CNNs), a class of deep neural networks (DNNs), have opened promising avenues for CTVI. CNNs can model complex, nonlinear relationships by processing two‐dimensional or three‐dimensional medical image inputs to make them applicable to generating CTVI. Recognizing these advantages, recent studies have addressed that practical limitation of ${\mathrm{CTVI}}_{4\mathrm{DCT}}^{\mathrm{DIR}}$ by proposing CTVI from 4DCT images using DNN models (${\mathrm{CTVI}}_{4\mathrm{DCT}}^{\mathrm{DNN}}$). Although the training targets remained dependent on DIR‐based ventilation imaging, the proposed model was trained using DIR‐derived ventilation images as reference labels and could estimate lung ventilation without requiring intermediate DIR steps during inference.

There is one practical limitation of 4DCT despite its usefulness in generating CTVI using DIR or DNN. Motion artifacts, including blurring, duplication, overlapping, and incomplete structures,^14^ can be a significant source of inaccuracy in the estimation of CTVI.^15^ Even if motion artifacts occur only locally, inaccuracies can be introduced throughout the entire lung function in CTVI due to the percentile process. Motion artifacts represent a practical limitation and also pose two fundamental problems. The first is patient‐dependent problem: respiratory patterns and conditions vary among individuals and are difficult for clinicians to fully control through technical means because stable breathing requires patient cooperation.^16^ The second is operator‐dependent problem: identifying and evaluating motion artifacts often rely on qualitative assessment rather than standardized, quantitative criteria,^14^ making consistent interpretation difficult. Consequently, the decision to repeat a scan can vary between operators. As a result of both patient‐ and operator‐dependent issues, motion artifacts are multifaceted problems that not only degrade image accuracy but also trigger broader systemic issues that compromise the overall reliability of CTVI. In this study, we developed a framework to generate CTVI from planning CT (PCT), and demonstrated its estimation performance by comparison with ${\mathrm{CTVI}}_{4\mathrm{DCT}}^{\mathrm{DIR}}$.

## 2 METHODS

### 2.1 Patient cohort

As shown in Table 1, the patient cohort included 102 patients with locally‐advanced non‐small cell lung cancer (NSCLC) who received thoracic radiotherapy between 2014 and 2023. The patients received 2.0 Gy fractions for a total of 60‐66 Gy using a 6‐MV or 10‐MV external photon beam. Patients whose PCT and 4DCT images were acquired on different days were excluded from the cohort due to the potential inaccuracy of coordinate transformation based solely on the DICOM Image Position (Patient) tag (0020,0032), as patient positioning on the couch varies between days.

**TABLE 1:** Patient characteristics.

		*N*	%
Gender	Male	86	84.3
	Female	16	15.7
T stage	T1	21	20.6
	T2	39	38.2
	T3	25	24.5
	T4	17	16.7
N stage	N0	15	14.7
	N1	25	24.5
	N2	43	42.2
	N3	19	18.6
COPD	Yes	50	49.0
	No	52	51.0
		Median (range)	
Age		71 (43‐84)	
PTV volume (cc)		344 (45‐1310)	

COPD = chronic obstructive pulmonary disease; PTV = planning target volume.

### 2.2 PCT images acquisition

The patient anatomies were scanned using either the Bright Speed 16 (GE Healthcare, Milwaukee, WI, USA) or the SOMATOM Definition Flash AS (Siemens Healthcare, Erlangen, Germany), with a pixel size <1.0 mm and a slice thickness <2.5 mm. Motion artifacts including blurring, duplicate, overlapping, and incomplete structures^14^ can result from baseline drift, frequency variation, amplitude changes, and additive random observation noise.^16^ In accordance with clinical guidelines, patients who exhibited tumor motion greater than 5 mm during the preliminary fluoroscopy session were scanned under free‐breathing conditions using an immobilization system with respiratory restriction.^17^ This approach was selected instead of a breath‐hold technique, which was often unfeasible in certain patients due to factors such as advanced age or the presence of chronic obstructive pulmonary disease (COPD). Respiratory restriction, recommended in clinical guidelines to prevent interplay effects, also secondarily contributed in this study to reducing motion‐induced discrepancies between the patient lung and its depiction on CT images,^18^ resulting in reduced inter‐patient variability of such discrepancies within our patient cohort. The PCT images were acquired while ensuring the absence of baseline drift, frequency variation, amplitude changes, and additive random observation noise using a real‐time position management (RPM) system (Varian Medical Systems, California, USA).

### $2.3\;{\mathrm{CTVI}}_{4\mathrm{DCT}}^{\mathrm{DIR}}$ generation

The 4DCT images comprised 10 respiratory phases sorted using the RPM system. Each 4DCT image was manually reviewed through a double‐check process by a medical physicist and a radiation oncologist, and cases exhibiting significant artifacts such as blurring, duplication, overlapping, or incomplete anatomical structures, as described by Yamamoto et al.,^14^ were excluded from the study. Peak‐inhalation images were spatially registered to a peak‐exhalation image using a nonparametric volume‐based DIR method with parameter settings that achieved a spatial accuracy of <1.3 mm^19^ implemented in Elastix open‐source image registration toolkit. Following DIR, the registered images were manually reviewed and excluded from the study through a double‐check process by a medical physicist and a radiation oncologist. The ventilation images were generated by quantifying image processing based on the HU metric, which is a relative measure of radiodensity derived from the concept that CT numbers represent a linear combination of air‐like and water‐like components^20^ (Equation 1).

(1) $$\frac{V_{in}-V_{ex}}{V_{ex}}=1000\frac{HU_{in}-HU_{ex}}{HU_{ex}\left(1000+HU_{in}\right)},$$

Here, $\;V_{in}$ and $V_{ex}$ denote the registered peak‐inhalation image and peak‐exhalation image, and $HU_{in}$ and $HU_{ex}$ denote the HU for these images, respectively. ${\mathrm{CTVI}}_{4\mathrm{DCT}}^{\mathrm{DIR}}$ were generated by converging all quantified function values into percentile values (0–1). Each ${\mathrm{CTVI}}_{4\mathrm{DCT}}^{\mathrm{DIR}}$ consisted of function values quantified with HU metrics and was smoothed using a mask‐preserving median filter with a width of 3 × 3 × 3 voxels employed in VAMPIRE study.^21^

### 2.3 Image preprocessing

The image preprocessing applied to the PCT images input to the prediction model and the ${\mathrm{CTVI}}_{4\mathrm{DCT}}^{\mathrm{DIR}}$ (label data) is described as follows. In each image, the lung volume was defined by excluding the mediastinal organs and the gross‐tumor‐volume (GTV) from the volume defined in the Eclipse treatment‐planning‐system (Varian Medical Systems, California, USA) using the HU values in the range [−999, −250]. Here, the exclusion of the mediastinal organs was performed in accordance with established guidelines.^22^ The voxel values in the lung volume in the PCT images were rescaled linearly using following scale: −1000 HU = 0 and −250 HU = 1, where voxels with ≥250 were replaced at 1. For the ${\mathrm{CTVI}}_{4\mathrm{DCT}}^{\mathrm{DIR}}$, the voxels in the lung volume that fell outside the range of the mean ± 3 standard deviations were replaced with the corresponding boundary values (i.e., the mean ± 3 standard deviation) after confirming that the voxels followed a normal distribution. Image processing that applies statistical criteria derived from standard deviation has been often adopted in functional lung imaging to mitigate non‐physiological artifacts.^21^, ^23^, ^24^ Then, the voxel values were normalized by the mean value rather than the median value because the outlier processing described above was performed. Finally, down sampled to 192 × 192 × 192, adopting the largest matrix size used between previous studies^12^, ^13^, ^25^ in each image.

### 2.4 DNN training

**FIGURE 1:**
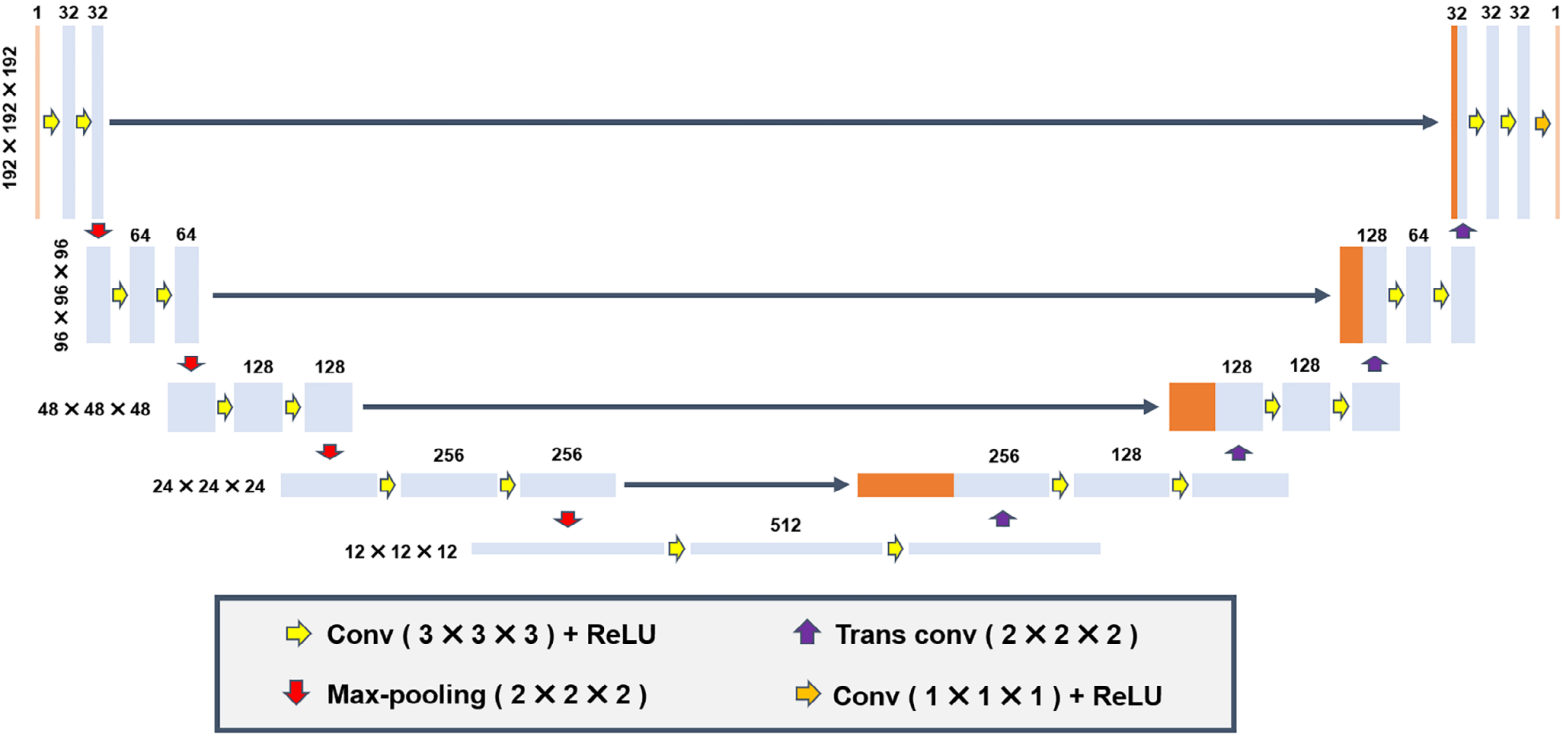
U‐net architecture employed in this study. Conv = convolutional layer; Trans conv = transposed convolutional layer.

The DNN‐based U‐net (Figure 1) takes a PCT image as input and an estimated pulmonary ventilation image (${\mathrm{CTVI}}_{\mathrm{PCT}}^{\mathrm{DNN}}$) as output. During training, ${\mathrm{CTVI}}_{4\mathrm{DCT}}^{\mathrm{DIR}}$ was used as the reference pulmonary ventilation image. This network is a slightly modified version that can import 192 × 192 × 192 volume data to estimate SPECT ventilation images from PCT images.^26^ An encoder with a depth of 4 and 5 resolution levels used 32, 64, 128, 256, and 512 feature channels at feature map sizes of 192 × 192 × 192, 96 × 96 × 96, 48 × 48 × 48, 24 × 24 × 24, and 12 × 12 × 12 voxels, respectively, resulting in a total of 18.3 million trainable parameters.

The encoding path comprises two 3 × 3 × 3 convolutions with a 1 × 1 × 1 stride and 1 × 1 × 1 padding, each of which is followed by a ReLU activation function and a 2 × 2 × 2 max pooling layer with a 2 × 2 × 2 stride. Note that the number of feature maps was doubled in each down‐sampling step. The decoding path comprises 2×2×2 transposed convolutions with a 2 × 2 × 2 stride, a skip connection with the feature maps with corresponding size from the encoding path, and two 3 × 3 × 3 convolutions with a 1 × 1 × 1 stride and 1 × 1 × 1 padding, each of which was followed by the ReLU activation function. The ${\mathrm{CTVI}}_{\mathrm{PCT}}^{\mathrm{DNN}}$ is output from a 1 × 1 × 1 convolutional layer with a stride of 1 × 1 × 1 followed by a ReLU activation function. This ReLU was adopted because pulmonary ventilation values are inherently non‐negative from a physiological perspective.

A model was trained using the Adam optimizer with a minibatch size of 4 to minimize the mean‐squared‐error (MSE) loss function. The model was trained for 200 epochs with an initial learning rate of 0.001, which was reduced by a factor of 10 every 20 epochs. Neither early stopping nor data augmentation was employed, and no hyperparameter search was performed. To evaluate the model performance robustly, a 5‐fold cross‐validation (CV) procedure was performed on 102 cases. In each iteration, one‐fold was used for testing and the remaining four folds were used for training, such that each case was included in the test set once across the five iterations.

### 2.5 Evaluation

The Spearman's r_s_, which indicates the degree of monotonicity, ranging from −1 to +1, where −1 represents a perfect negative correlation and +1 represents a perfect positive correlation, was calculated between the ${\mathrm{CTVI}}_{\mathrm{PCT}}^{\mathrm{DNN}}$ and ${\mathrm{CTVI}}_{4\mathrm{DCT}}^{\mathrm{DIR}}$ using Equation (2).

(2) $$r_{s}=1\;-\;\frac{6\sum_{i=1}^{N}D_{i}^{2}\;}{N^{3}-N}$$

Here, $D_{i}$ denotes the difference between the ranks of the values at voxel i in the reference and estimated images, and N denotes the total number of voxels in each image.

The Dice similarity coefficient (DSC), which indicates the geometric similarity between ${\mathrm{CTVI}}_{\mathrm{PCT}}^{\mathrm{DNN}}$ and ${\mathrm{CTVI}}_{4\mathrm{DCT}}^{\mathrm{DIR}}$ with respect to the functional regions, was calculated for three functional lung regions according to the proposed functional avoidance radiotherapy,^1^ that is, the highest 33.3%, the intermediate 33.3%, and the lowest 33.3%, using Equation (3), corresponding to DSC_high_, DSC_moderate_, and DSC_low_, respectively.

(3) $$DSC\;\left(a,\;b\right)=\frac{2\left|a\cap b\right|}{\left|a\right|+\left|b\right|}$$

Here, a and b denote the volumes of the functional lung regions in ${\mathrm{CTVI}}_{\mathrm{PCT}}^{\mathrm{DNN}}$ and ${\mathrm{CTVI}}_{4\mathrm{DCT}}^{\mathrm{DIR}}$, respectively. In this study, image preprocessing, DNN development, training, and testing were performed using MATLAB (MathWorks, Natick, Massachusetts, USA) with an NVIDIA RTX A6000 graphics processing unit with 48 GB memory.

## 3 RESULTS

Between 2014 and 2023, in addition to the patient cohort of 102 patients (Table 1), seven patients scanned on different days were identified due to the presence of motion artifacts even in their rescanned 4DCT images. No patient in this study required a repeat scan due to baseline drift, frequency variation, amplitude changes, and additive random observation noise during PCT acquisition. Table 2 shows Spearman's r_s_ and DSC results for DSC_high_, DSC_moderate_, and DSC_low_ obtained between ${\mathrm{CTVI}}_{4\mathrm{DCT}}^{\mathrm{DIR}}$ and ${\mathrm{CTVI}}_{\mathrm{PCT}}^{\mathrm{DNN}}$ for the testing set (*n* = 31) every CV fold. The average similarity metrics (±SD) for each fold between ${\mathrm{CTVI}}_{\mathrm{PCT}}^{\mathrm{DNN}}$ and ${\mathrm{CTVI}}_{4\mathrm{DCT}}^{\mathrm{DIR}}$ were 0.77 ± 0.08, 0.68 ± 0.06, 0.51 ± 0.08, and 0.75 ± 0.04 for Spearman's r_s_, DSC_high_, DSC_moderate_, and DSC_low_, respectively.

**TABLE 2:** Estimation performance of ${\mathrm{CTVI}}_{\mathrm{PCT}}^{\mathrm{DNN}}.$

		DSC		
Fold no.	Spearman's r_s_	High	Moderate	Low
1	0.77 ± 0.08	0.68 ± 0.07	0.51 ± 0.07	0.76 ± 0.05
2	0.75 ± 0.09	0.67 ± 0.08	0.50 ± 0.07	0.74 ± 0.06
3	0.78 ± 0.06	0.69 ± 0.06	0.52 ± 0.07	0.76 ± 0.04
4	0.78 ± 0.08	0.69 ± 0.07	0.52 ± 0.07	0.75 ± 0.05
5	0.78 ± 0.07	0.69 ± 0.07	0.53 ± 0.07	0.77 ± 0.04
6	0.78 ± 0.09	0.69 ± 0.08	0.52 ± 0.08	0.76 ± 0.05
7	0.77 ± 0.08	0.68 ± 0.06	0.52 ± 0.07	0.75 ± 0.05
8	0.76 ± 0.09	0.67 ± 0.08	0.51 ± 0.08	0.76 ± 0.05
9	0.76 ± 0.09	0.68 ± 0.08	0.51 ± 0.08	0.75 ± 0.05
10	0.77 ± 0.09	0.68 ± 0.07	0.51 ± 0.08	0.75 ± 0.06

${\mathrm{CTVI}}_{\mathrm{PCT}}^{\mathrm{DNN}}$ = CT ventilation image from PCT based on DNN; DSC = dice similarity coefficient.

**FIGURE 2:**
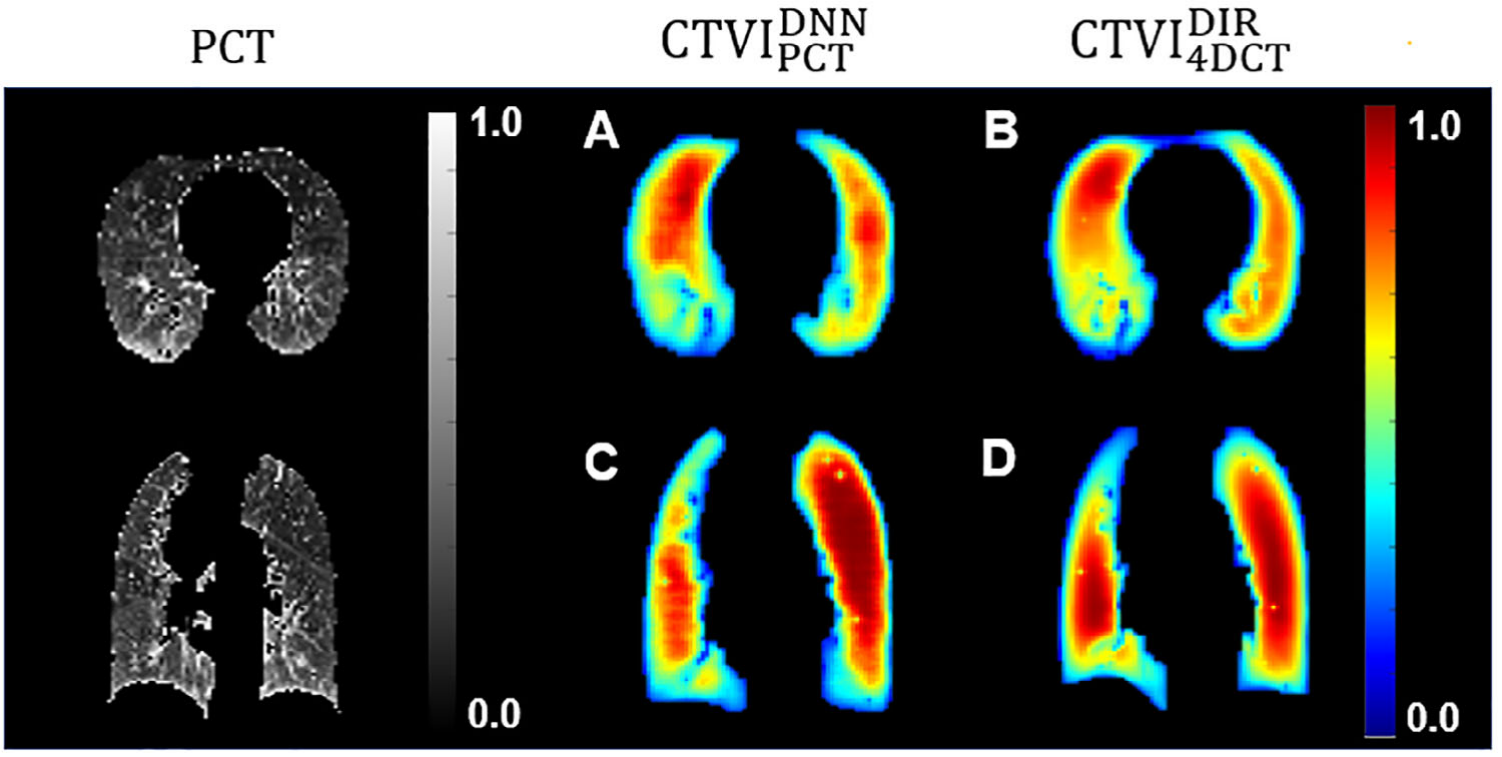
Representative patient showing good performance of PCT images, CTVI_PCT^DNN, and CTVI_4DCT^DIR in transverse and coronal slices. The color scale represents unitless percentile values (0–1), obtained by converting voxel values to percentiles within each patient.$\;{\mathrm{CTVI}}_{\mathrm{PCT}}^{\mathrm{DNN}}$ = CT ventilation image from PCT based on DNN; ${\mathrm{CTVI}}_{4\mathrm{DCT}}^{\mathrm{DIR}}$ = CT ventilation image from 4DCT.

**FIGURE 3:**
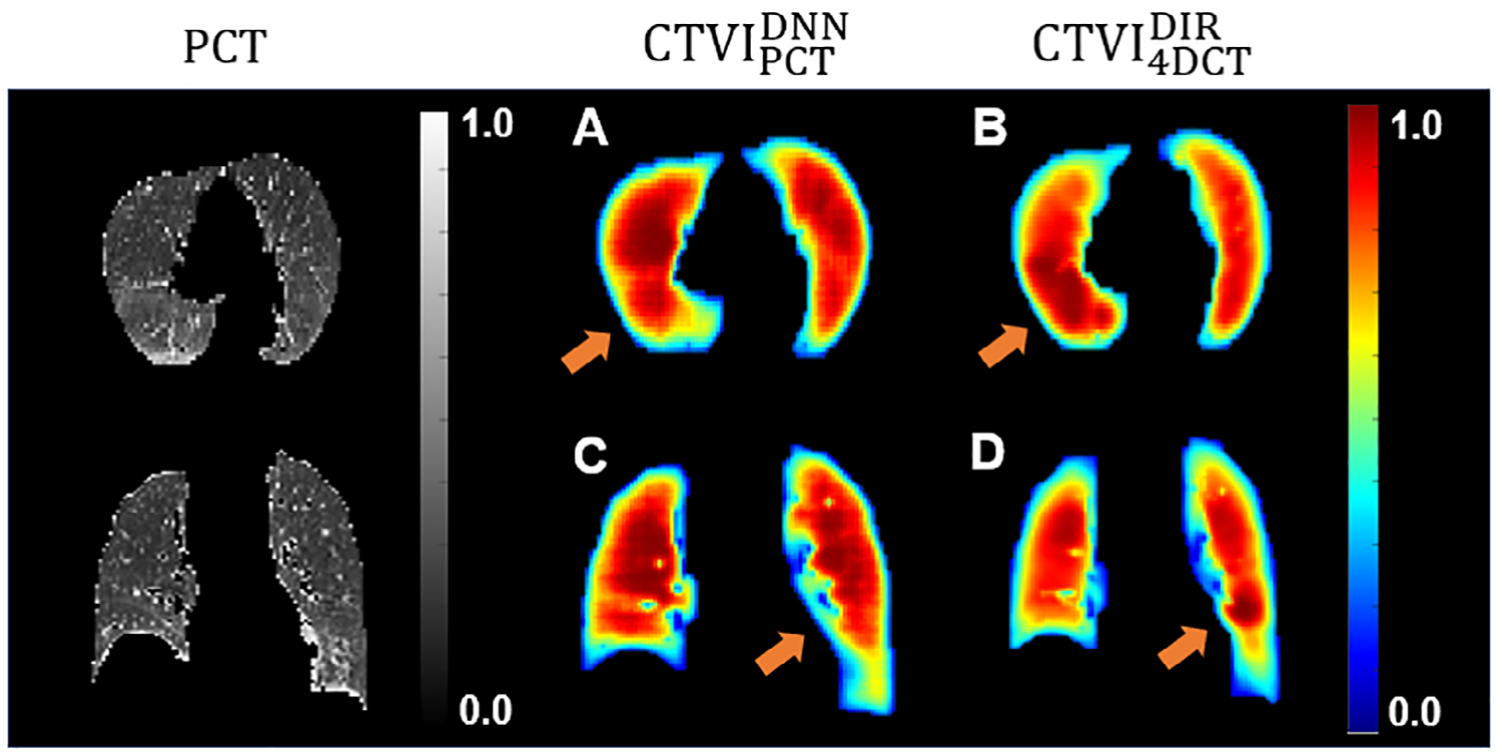
Representative patient showing moderate performance of PCT images, ${\mathrm{CTVI}}_{\mathrm{PCT}}^{\mathrm{DNN}}$, and ${\mathrm{CTVI}}_{4\mathrm{DCT}}^{\mathrm{DIR}}$ in transverse and coronal slices. The color scale represents unitless percentile values (0–1), obtained by converting voxel values to percentiles within each image and patient.$\;{\mathrm{CTVI}}_{\mathrm{PCT}}^{\mathrm{DNN}}$ = CT ventilation image from PCT based on DNN; ${\mathrm{CTVI}}_{4\mathrm{DCT}}^{\mathrm{DIR}}$ = CT ventilation image from 4DCT.

**FIGURE 4:**
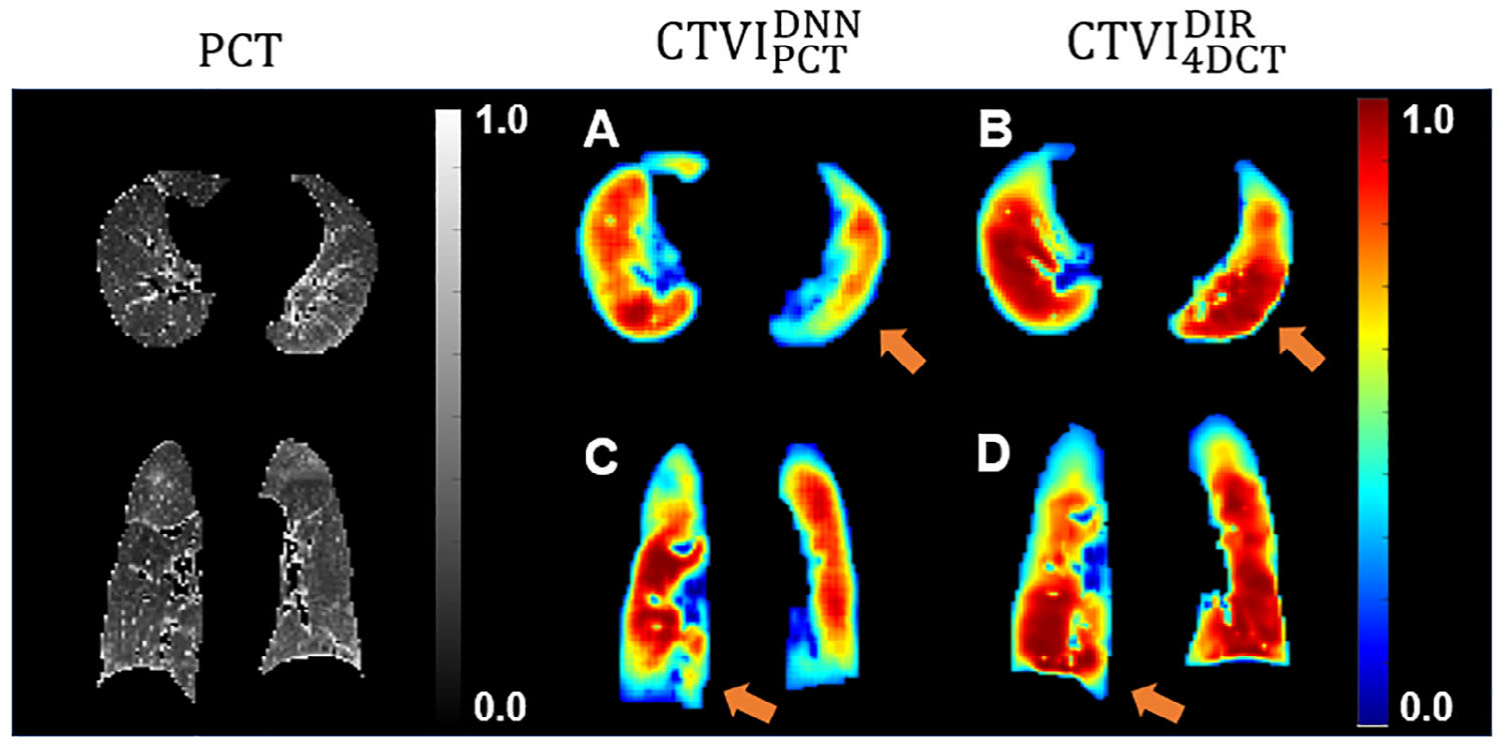
Representative patient showing poor performance of PCT images, ${\mathrm{CTVI}}_{\mathrm{PCT}}^{\mathrm{DNN}}$, and ${\mathrm{CTVI}}_{4\mathrm{DCT}}^{\mathrm{DIR}}$ in transverse and coronal slices. The color scale represents unitless percentile values (0–1), obtained by converting voxel values to percentiles within each image and patient.$\;{\mathrm{CTVI}}_{\mathrm{PCT}}^{\mathrm{DNN}}$ = CT ventilation image from PCT based on DNN; ${\mathrm{CTVI}}_{4\mathrm{DCT}}^{\mathrm{DIR}}$ = CT ventilation image from 4DCT.

Figures 2, 3, 4 show the PCT images and the ${\mathrm{CTVI}}_{\mathrm{PCT}}^{\mathrm{DNN}}$ and ${\mathrm{CTVI}}_{4\mathrm{DCT}}^{\mathrm{DIR}}$ results for representative patients, respectively. These figures show three comparisons between ${\mathrm{CTVI}}_{\mathrm{PCT}}^{\mathrm{DNN}}$ and ${\mathrm{CTVI}}_{4\mathrm{DCT}}^{\mathrm{DIR}}$, demonstrating relatively good agreement (Figure 2), moderate agreement (Figure 3), and poor agreement (Figure 4). In Figure 2, the correlation between ${\mathrm{CTVI}}_{\mathrm{PCT}}^{\mathrm{DNN}}$ (panels A and C) and ${\mathrm{CTVI}}_{4\mathrm{DCT}}^{\mathrm{DIR}}$, (panels B and C) with r_s_ = 0.91, DSC_high_ = 0.83, DSC_moderate_ = 0.70, and DSC_low_ = 0.84 demonstrates that only minor local discrepancies are observed in this representative patient with relatively high r_s_ and DSC values. A patient with moderate agreement, that is, r_s_ = 0.76, DSC_high_ = 0.66, DSC_moderate_ = 0.48, and DSC_low_ = 0.75, is shown in Figure 3. In this patient, regions that have functional values approximately 0.7, as indicated by the orange arrows in panels A and C, should be displayed with values of close to 1.0 in panels B and D, indicating insufficient emphasis on high‐functioning regions. Figure 4 shows a representative patient with poor agreement, that is, r_s_ = 0.65, DSC_high_ = 0.61, DSC_moderate_ = 0.47, and DSC_low_ = 0.69. Here, regions with low functional values (<0.4), as demonstrated by the orange arrows in panels A and C, should be displayed as high functional values (>0.7) over a relatively broad extent in panels B and D.

## 4 DISCUSSION

We developed a framework to estimate ventilation images from PCT images, and we demonstrated its estimation performance in terms of Spearman's r_s_ and DSC on locally‐advanced NSCLC patients. The proposed network estimates CTVI based on the HU metric rather than a Jacobian metric because the HU metric yields superior predictive performance.^27^ In our study, the Spearman's r_s_ of 0.77 ± 0.08 exceeded the commonly accepted threshold of 0.7 to indicate a strong association. In contrast the DSC cutoff range of 0.8 < DSC ≤ 1.0, proposed by Yamamoto et al.^28^ indicating perfect agreement, was not satisfied our 0.68 ± 0.06 for DSC_high_, 0.51 ± 0.08 for DSC_moderate_, and 0.75 ± 0.04 for DSC_low_.

Direct comparison of the reference ${\mathrm{CTVI}}_{4\mathrm{DCT}}^{\mathrm{DIR}}$ with SPECT, the standard modality for evaluating lung function, was not possible because of the retrospective study design and the absence of SPECT data in our patient cohort. Therefore, the lack of comparison with SPECT remains a limitation of the present study. In a study by Yamamoto et al., significant correlations between ${\mathrm{CTVI}}_{4\mathrm{DCT}}^{\mathrm{DIR}}$ and SPECT were demonstrated through direct comparison of the two modalities.^7^ Notably, the accuracy of ${\mathrm{CTVI}}_{4\mathrm{DCT}}^{\mathrm{DIR}}$ may be influenced by image quality and the accuracy of DIR.^32^ We attempted to minimize inaccuracies in ${\mathrm{CTVI}}_{4\mathrm{DCT}}^{\mathrm{DIR}}$ by adopting DIR parameters reported in a previous study^19^ and applying the mask‐preserving median filter with a kernel size of 3 × 3 × 3 voxels used in the VAMPIRE study.^21^

The lower DSC_moderate_ is attributable to the threshold‐based definition of the functional regions. The thresholds used to define functional regions vary depending on estimation errors in voxel values. Moderate‐functional regions were defined using two thresholds, whereas high‐functional and low‐functional regions were defined using a single lower or upper threshold. Consequently, moderate‐functional regions are more susceptible to uncertainty than high‐functional and low‐functional regions, resulting in lower DSC_moderate_. A similarly lower DSC_moderate_ has also been reported previously.^26^ The clinical rationale for delineating the three functional regions into approximately equal volumes is based on the concept of functional‐lung‐avoidance radiation therapy (FLAR).^1^ In FLAR, sparing high‐functional lung regions is prioritized, whereas irradiation of low‐functional lung regions is considered relatively more acceptable. In contrast, moderate‐functional regions lie between high‐ and low‐functional regions and therefore have a less distinct role in FLAR. Consequently, the clinical impact of the lower DSC_moderate_ relative to DSC_high_ and DSC_low_ may be limited. However, in patients for whom adequate sparing of high‐functional lung regions is difficult due to tumor size, tumor extent, or competing OAR constraints, moderate‐functional regions may assume a more important role in functional‐lung‐avoidance radiotherapy.

Zhen et al. reported a Spearman's r_s_ of 0.65 and a DSC values the highest 50% function value of 0.41 between ${\mathrm{CTVI}}_{\mathrm{PCT}}^{\mathrm{DNN}}$ and ${\mathrm{CTVI}}_{4\mathrm{DCT}}^{\mathrm{DIR}}$ based on the Jacobian metric.^12^ The discrepancies between our results and those reported by Zhen et al. are potentially attributed to two factors: the use of different metrics and the inclusion of outliers in the label data, which could hinder the optimization of the network's hyperparameters. In contrast, we replaced the ${\mathrm{CTVI}}_{4\mathrm{DCT}}^{\mathrm{DIR}}$ voxel values outside the range of the mean ± 3 SD with the corresponding boundary values. In addition, our dataset was larger than theirs, which is expected to yield better performance in terms of data diversity and a reduced risk of overfitting.

The predicted ventilation images display only a part of the lung function displayed in the ${\mathrm{CTVI}}_{4\mathrm{DCT}}^{\mathrm{DIR}}$. Although PCT images were acquired using the RPM system, some motion artifacts remained unavoidable. DIBH CT images may further reduce these artifacts. However, similar to PCT images, DIBH CT images primarily depicts patient anatomy, with only minimal anatomical changes resulting from respiratory motion. The presence of lung function that can be predicted from patient anatomy has been suggested by De Backer et al. using computed fluid dynamics^29^ and by Chen et al. using a super‐voxel‐based technique.^30^ Park et al. reported associations between CT density measurements and pulmonary function parameters, including the diffusing capacity of the lungs for carbon monoxide (DLCO) and forced expiratory volume in 1 s (FEV1).^31^ Furthermore, CT images have been successfully used to estimate functional lung images derived from SPECT using deep learning.^26^ This finding suggests that the neural network may learn CT image features associated with lung function rather than relying primarily on respiratory motion information.

Several limitations related to our patient cohort should be acknowledged. First, because the patient cohort was derived from a single institution, the possibility of overfitting cannot be excluded, and the generalizability of the model remains to be established. To reduce the risk of overfitting, we performed CV in the present study. The use of multi‐institutional patient cohorts could further reduce the risk of overfitting and enable external testing using independent patient data to assess model generalizability. Second, because cases with substantial 4DCT artifacts or DIR errors were excluded, the patient cohort may have been biased toward patients with higher‐quality respiratory motion or imaging data. Although this approach provided a cleaner reference standard, it limits the generalizability of the proposed method to patients who would otherwise be excluded from clinically acceptable ${\mathrm{CTVI}}_{4\mathrm{DCT}}^{\mathrm{DIR}}$ analysis. Future studies are warranted to evaluate the applicability of the proposed approach in multi‐institutional cohorts, including this clinically important patient population.

It is important to note that the ${\mathrm{CTVI}}_{\mathrm{PCT}}^{\mathrm{DNN}}$ does not imply treatment of locally‐advanced NSCLC without 4DCT image acquisition. In thoracic radiotherapy, the internal target volume (ITV), which represents the volume encompassing all possible locations of the GTV throughout the respiratory cycle, recommended to be determined using 4DCT images.^17^ This approach can ensure that the radiation beam effectively targets the tumor, even as it moves with respiration. Even if motion artifacts arise in regions unrelated to ITV determination, they can lead to the exclusion of the patients from the generation of clinically acceptable ${\mathrm{CTVI}}_{4\mathrm{DCT}}^{\mathrm{DIR}}$, because motion artifacts can affect the overall accuracy of this image when percentile processing is applied. ${\mathrm{CTVI}}_{\mathrm{PCT}}^{\mathrm{DNN}}$ may provide physiological information about regional lung function for such patients from PCT images acquired while ensuring variations in respiratory frequency and amplitude, as well as additive random observation noise. Further investigation is essential to quantify potential inaccuracies in the physiological information about regional lung function provided by ${\mathrm{CTVI}}_{\mathrm{PCT}}^{\mathrm{DNN}}$.

There is potential to improve the estimation performance of CTVI from PCT images acquired with breath‐hold using DNN. Daniel's study demonstrated that even with high‐speed helical CT scan, free‐breathing acquisition resulted in a significant discrepancy between the patient anatomy and its depiction in the images, which was observed as motion artifacts such as blurring.^32^ These results indicate that such discrepancies were not observed as obvious artifacts in our patient cohort, but they can still inherently exist at a level below that of overt artifacts. Since a part of the ventilation features displayed on ${\mathrm{CTVI}}_{4\mathrm{DCT}}^{\mathrm{DIR}}$ can be captured from the patient anatomy as demonstrated in this study, CTVI from PCT acquired with breath‐hold, which can represent the patient anatomy more accurately, is expected to better capture these. One problem is that a subset of patients with locally‐advanced NSCLC are unable to undergo CT images acquisition under breath‐hold conditions. A clinical trial of DIBH‐based radiotherapy on locally‐advanced NSCLC reported that 13% of patients were unable to perform breath‐hold adequately.^33^ In our patient cohort, PCT images acquired with free‐breathing conditions imposed no patient‐related limitations for the estimation of ventilation images, indicating that ventilation features for nearly all locally‐advanced NSCLC patients are can be displayed by ${\mathrm{CTVI}}_{\mathrm{PCT}}^{\mathrm{DNN}}$. If the estimation performance of CTVI from PCT images acquired with breath‐hold is demonstrated, a new strategy such as using breath‐hold PCT as the first choice and employing ${\mathrm{CTVI}}_{\mathrm{PCT}}^{\mathrm{DNN}}$ for patients unable to undergo CT images acquisition under breath‐hold conditions could be proposed. Further studies are required to advance CTVI estimation using DNN.

## 5 CONCLUSION

To address the growing clinical need for effective strategies for thoracic radiotherapy, this paper has proposed a framework for ${\mathrm{CTVI}}_{\mathrm{PCT}}^{\mathrm{DNN}}$ and demonstrated its estimation performance on locally‐advanced NSCLC patients by comparison with ${\mathrm{CTVI}}_{4\mathrm{DCT}}^{\mathrm{DIR}}$. In addition to being high‐resolution and not requiring additional costs compared with ${\mathrm{CTVI}}_{4\mathrm{DCT}}^{\mathrm{DIR}}$, ${\mathrm{CTVI}}_{\mathrm{PCT}}^{\mathrm{DNN}}$ may provide physiological information about regional lung function for patients excluded from the generation of clinically acceptable ${\mathrm{CTVI}}_{4\mathrm{DCT}}^{\mathrm{DIR}}$. Further investigation is essential to quantify potential inaccuracies in the physiological information about regional lung function provided by ${\mathrm{CTVI}}_{\mathrm{PCT}}^{\mathrm{DNN}}$.

## AUTHOR CONTRIBUTIONS

All authors contributed to the writing and editing of the article, and approved the final version.

## FUNDING INFORMATION

This work was supported by the JSPS KAKENHI 24K18818.

## CONFLICT OF INTEREST STATEMENT

The authors declare no conflicts of interest.

## ETHICS STATEMENT

2024‐1‐602

## Data Availability

The data that support the findings of this study are avail‐able from the corresponding author upon reasonable request.
